# Continuous epidural saline infusion combined with intranasal dexmedetomidine for severe post-dural puncture headache with intracranial hypotension: a case report

**DOI:** 10.3389/fmed.2026.1929098

**Published:** 2026-10-26

**Authors:** Yinghong Li, Qingle Kang, Huiyan Liang, Haiyan Zhang, Shengjie Chen, Xiangyi Lin, Haobin Peng

**Affiliations:** 1 Department of Anesthesiology, Shunde Women and Children’s Hospital of Guangdong Medical University, Foshan, Guangdong, China; 2 Department of Anesthesiology, Guangzhou University of Traditional Chinese Medicine ShunDe Traditional Chinese Medicine Hospital, Foshan, Guangdong, China

**Keywords:** case report, dexmedetomidine, epidural injection, intracranial hypotension, post-dural puncture headache

## Abstract

**Background:**

Post-dural puncture headache (PDPH) is a common complication of obstetric neuraxial anesthesia. Epidural blood patch (EBP) is the standard treatment for moderate-to-severe PDPH, but it may be deferred or relatively contraindicated when systemic infection is suspected. Current evidence on alternative treatments for PDPH has mainly focused on single interventions, and reports describing combined therapeutic strategies remain limited.

**Case presentation:**

A 37-year-old parturient developed accidental dural puncture during epidural labor analgesia and subsequently experienced severe PDPH 2 h after delivery, with a sitting numerical rating scale (NRS) score of 10. Cranial magnetic resonance imaging (MRI) showed features consistent with intracranial hypotension, including narrowing of the suprasellar cistern and inferior displacement of the cerebellar tonsils. Because high fever (39.2 °C) and leukocytosis raised concern for possible systemic infection, EBP was considered high risk. Continuous epidural normal saline infusion was administered through the retained epidural catheter at 10 mL/h, together with empirical ceftriaxone, non-steroidal analgesia, bed rest, and fluid support. Intranasal dexmedetomidine 50 μg per dose twice daily was added at approximately 24 h postpartum because of persistent headache. Within 48 h, the patient's headache improved in a stepwise manner: the sitting NRS score was 10 at 24 h postpartum, 4 at 36 h postpartum, and 0 at 48 h postpartum. Follow-up MRI on postpartum day 5 showed marked improvement in the signs of intracranial hypotension. No recurrence occurred at 1- and 3-month follow-up.

**Conclusion:**

In selected patients with severe PDPH for whom EBP is unsuitable or high risk, continuous epidural saline infusion combined with intranasal dexmedetomidine may be a feasible non-blood-patch alternative, although causality cannot be established from a single case because of concurrent interventions and possible spontaneous improvement. Larger studies are required to evaluate safety, efficacy, and optimal dosing.

## Highlights

1. To our knowledge, this may be among the first reports describing successful treatment of severe PDPH with intracranial hypotension using combined continuous epidural saline infusion and intranasal dexmedetomidine in a patient for whom EBP was considered high risk.
2. MRI confirmed intracranial hypotension with characteristic imaging findings, which are not consistently documented in published PDPH case reports.

## Introduction

Post-dural puncture headache (PDPH) is a frequent complication of neuraxial anesthesia and is particularly relevant in obstetric patients (1). It is mainly attributed to persistent cerebrospinal fluid (CSF) leakage after dural puncture, which can reduce CSF pressure and produce an orthostatic headache. Although many cases improve with conservative measures, severe or persistent PDPH may impair postpartum recovery, breastfeeding, sleep, and maternal care of the newborn, and can be associated with longer-term headache, back pain, and functional disability (2–4). Epidural blood patch (EBP) remains the standard treatment for moderate-to-severe PDPH that fails to respond to conservative therapy, with reported success rates of approximately 70%–98% (5). However, EBP is invasive and may cause complications including repeat dural puncture, infection, pneumocephalus, and neuraxial hematoma (6). In patients with fever, suspected bacteremia, local infection, or coagulopathy, EBP may be contraindicated or deferred (7). Therefore, non-blood-patch strategies are clinically important when EBP is considered unsafe.

Epidural saline injection or continuous saline infusion acts near the dural defect and may reduce CSF leakage by increasing epidural pressure. This pressure effect may bring the dura and arachnoid closer together, temporarily reduce leakage through the puncture site, and create conditions favorable for spontaneous dural healing (8). Che et al. retrospectively studied 87 patients with accidental dural puncture and reported that continuous epidural saline infusion shortened the duration of PDPH and reduced its incidence (9). A recent systematic review and meta-analysis by Jing et al. also suggested that intrathecal or epidural normal saline after dural puncture was associated with a reduced incidence of PDPH (RR = 0.57) and EBP requirement (RR = 0.37) (10). These findings indicate that epidural saline infusion may be a simple and relatively safe option for PDPH prevention or treatment.

Dexmedetomidine, a highly selective α2-adrenergic receptor agonist, has recently shown potential benefit in PDPH treatment, possibly through cerebral vasoconstriction and sedative-analgesic effects (11, 12). Kumar et al. reported that nebulized dexmedetomidine was superior to fentanyl and placebo in reducing PDPH pain scores and the need for rescue analgesia (13). Thomas et al. described two obstetric cases in which nebulized dexmedetomidine was associated with rapid and complete relief of PDPH (14). In addition to nebulization, dexmedetomidine can be absorbed through the nasal mucosa; intranasal administration has been reported to have a bioavailability of approximately 59%, and it is relatively convenient and acceptable to patients (15).

Current research on non-EBP treatments for PDPH has mainly evaluated single interventions. The combined use of epidural saline infusion and intranasal dexmedetomidine, which may act through complementary mechanical and pharmacological mechanisms, has rarely been described. We report a case of severe PDPH with MRI-confirmed intracranial hypotension after accidental dural puncture in a parturient for whom EBP was considered high risk because of fever and suspected infection. The patient was successfully treated with continuous epidural saline infusion through a retained epidural catheter combined with intranasal dexmedetomidine. As far as we know, this is among the first reports of such a combinatorial therapy regimen.

## Case report

Written informed consent for publication was obtained from the patient before manuscript preparation.

### General condition and preoperative preparation

A 37-year-old woman at 39 weeks of gestation was admitted for planned vaginal delivery because of gestational diabetes mellitus. She had no history of migraine, chronic headache, neurological disease, coagulation disorder, anticoagulant use, or previous neuraxial anesthesia. Routine preprocedural laboratory tests showed no clinically significant abnormality. She was classified as American Society of Anesthesiologists physical status II and elected epidural labor analgesia after informed consent.

### Accidental dural puncture and labor

At 7:00 a.m., an anesthesiologist attempted epidural puncture at the L3–4 interspace for labor analgesia. During the procedure, the dura was unintentionally punctured, and clear CSF was observed through the needle. The needle was gradually withdrawn until CSF could no longer be aspirated, after which loss of resistance to saline was obtained, confirming epidural placement. Twenty milliliters of normal saline was then injected into the epidural space, and an epidural catheter was advanced to a depth of 5 cm. Aspiration through the catheter yielded neither CSF nor blood. A 3-mL test dose of 1% lidocaine produced no evidence of subarachnoid block; subsequently, a 10-mL loading dose of 0.1% ropivacaine with 0.5 μg/mL sufentanil was administered. Continuous epidural infusion of the same solution was then initiated at 10 mL/h, with patient-controlled epidural analgesia available as needed. During labor, the patient's vital signs remained stable, her blood pressure was normal, and she reported no headache, nausea, or abnormal sensory or motor symptoms. She delivered a healthy full-term infant vaginally, with Apgar scores of 9 and 10 at 1 and 5 min, respectively.

### Combined therapy and follow-up

The subsequent clinical course is summarized in Table 1.

**Table 1:** Clinical course after delivery and treatment response.

Time point	Symptoms/NRS	Investigations	Management and outcome
2 h postpartum	Bilateral temporal orthostatic headache; sitting NRS 10, supine NRS 2–3; no focal deficit.	T 36.9 °C; examination otherwise unremarkable; BP 120/75 mmHg, HR 91 bpm.	PDPH diagnosed; bed rest and IV fluids started.
Afternoon, day 0	Headache worsened; mild neck stiffness.	Tmax 39.2 °C; WBC 18.39 × 10^9^/L; neutrophils 17.25 × 10^9^/L; CRP 2.4 mg/L; PCT < 0.05 ng/mL; MRI consistent with intracranial hypotension; BP 112/75 mmHg, HR 80 bpm.	EBP deferred because systemic infection could not be excluded；Epidural saline 10 mL/h; ceftriaxone, diclofenac, bed rest, IV fluids.
12 h postpartum	Supine NRS 2; sitting NRS 10; poor sleep.	Tmax 38.0 °C; BP 100/60 mmHg, HR 78 bpm.	Epidural saline 10 mL/h; ceftriaxone, diclofenac, bed rest, IV fluids.
24 h postpartum	Supine NRS 2; sitting NRS 10.	No new abnormal finding; BP 109/68 mmHg, HR 75 bpm; T 37.5 °C.	The treatment regimen was continued; additional intranasal dexmedetomidine 50 μg per dose twice daily; sleep improved.
36 h postpartum	Supine NRS 2; sitting NRS 4.	No new abnormal finding; BP 120/73 mmHg, HR 82 bpm; T 38.0 °C.	Second dose of intranasal dexmedetomidine given; combined treatment continued; stepwise headache relief.
48 h postpartum	Supine and sitting NRS 0; tolerated sitting at 45° and 90° for 5 min each.	T 37.0 °C; CRP 40.1 mg/L; PCT <0.05 ng/mL; BP 125/63 mmHg, HR 85 bpm.	Epidural catheter removed after symptom resolution; sleep improved; intranasal dexmedetomidine discontinued because the patient was headache-free; only two doses (within 24 h) had been administered.
Postpartum day 3	No spontaneous headache.	WBC 12.11 × 10^9^/L; CRP 30.8 mg/L; PCT <0.05 ng/mL; BP 130/80 mmHg, HR 69 bpm; T 37.2 °C.	Supportive care; inflammatory markers improved.
Postpartum day 5–6; 1 and 3 months	No discomfort during ambulation; meningeal signs negative. No recurrence at follow-up.	Day 5 MRI improved; clinical status stable.	Discharged on day 6 without headache; no recurrence or related complications were reported at 1- and 3-month follow-up.

NRS, numerical rating scale; PDPH, post-dural puncture headache; IV, intravenous; WBC, white blood cell; CRP, C-reactive protein; PCT, procalcitonin; MRI, magnetic resonance imaging; EBP, epidural blood patch; BP, blood pressure; HR, heart rate; T, temperature.

Approximately two hours after delivery, at 11:00 a.m., the patient suddenly developed bilateral temporal tearing-type headache. She was alert and had no nausea or vomiting. The headache was strongly postural: it worsened markedly when she slowly sat upright (NRS 10) and was substantially relieved when she lay supine (NRS 2–3). Physical examination showed blood pressure of 120/75 mmHg, heart rate of 91 beats/min, and body temperature of 36.9 °C. Auditory and abducens nerve examinations were normal. The neck was supple without resistance, meningeal irritation signs were negative, limb muscle strength was grade V, anal sphincter function was intact, and the lumbar puncture site showed no erythema, swelling, leakage, or tenderness. PDPH was clinically diagnosed. Strict bed rest and intravenous fluid support of 2,000–2,500 mL/day were initiated. The clinical timeline is shown in Figure 1.

**Figure 1:**
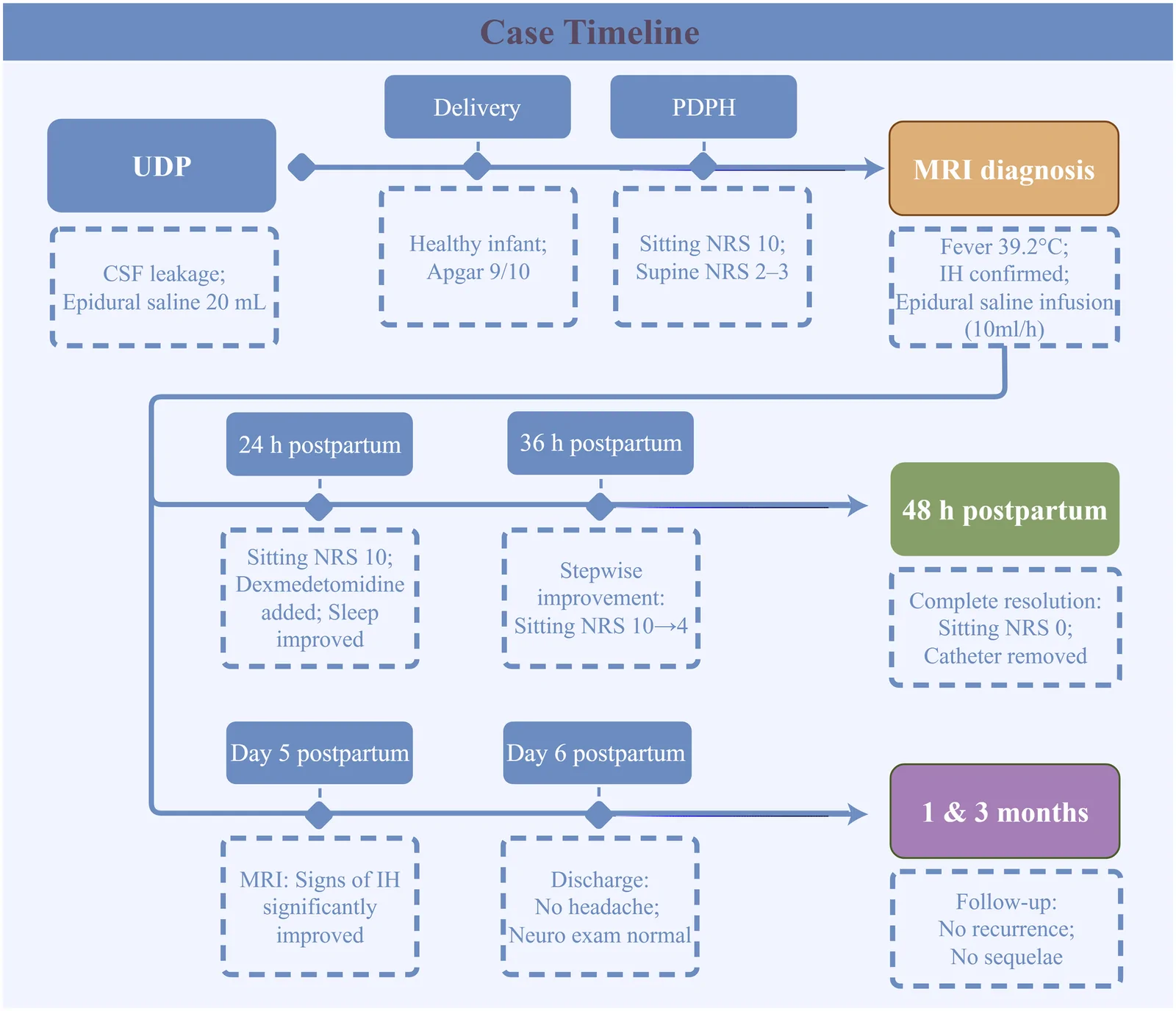
Clinical timeline of the case. UDP, unintentional dural puncture; PDPH, post-dural puncture headache; NRS, numerical rating scale; IH, intracranial hypotension; CSF, cerebrospinal fluid; MRI, magnetic resonance imaging. The figure was created by Figdraw (https://www.figdraw.com).

That afternoon, the temperature rose to 39.2 °C and the headache worsened, with an NRS score of 10. Mild neck stiffness was present, with a chin-to-sternum distance of approximately 20 cm. Emergency cranial MRI showed narrowing of the suprasellar cistern (approximately 1.9 mm; normal ≥4 mm), narrowing of the mamillopontine distance (approximately 5.8 mm; normal ≥6.5 mm), inferior displacement of the cerebellar tonsils toward the foramen magnum, and mild venous sinus dilatation. These findings were consistent with intracranial hypotension (Figure 2; Table 2). Laboratory tests showed a white blood cell count of 18.39 × 10^9^/L, neutrophil count of 17.25 × 10^9^/L, C-reactive protein (CRP) level of 2.4 mg/L, and procalcitonin <0.05 ng/mL.

**Figure 2:**
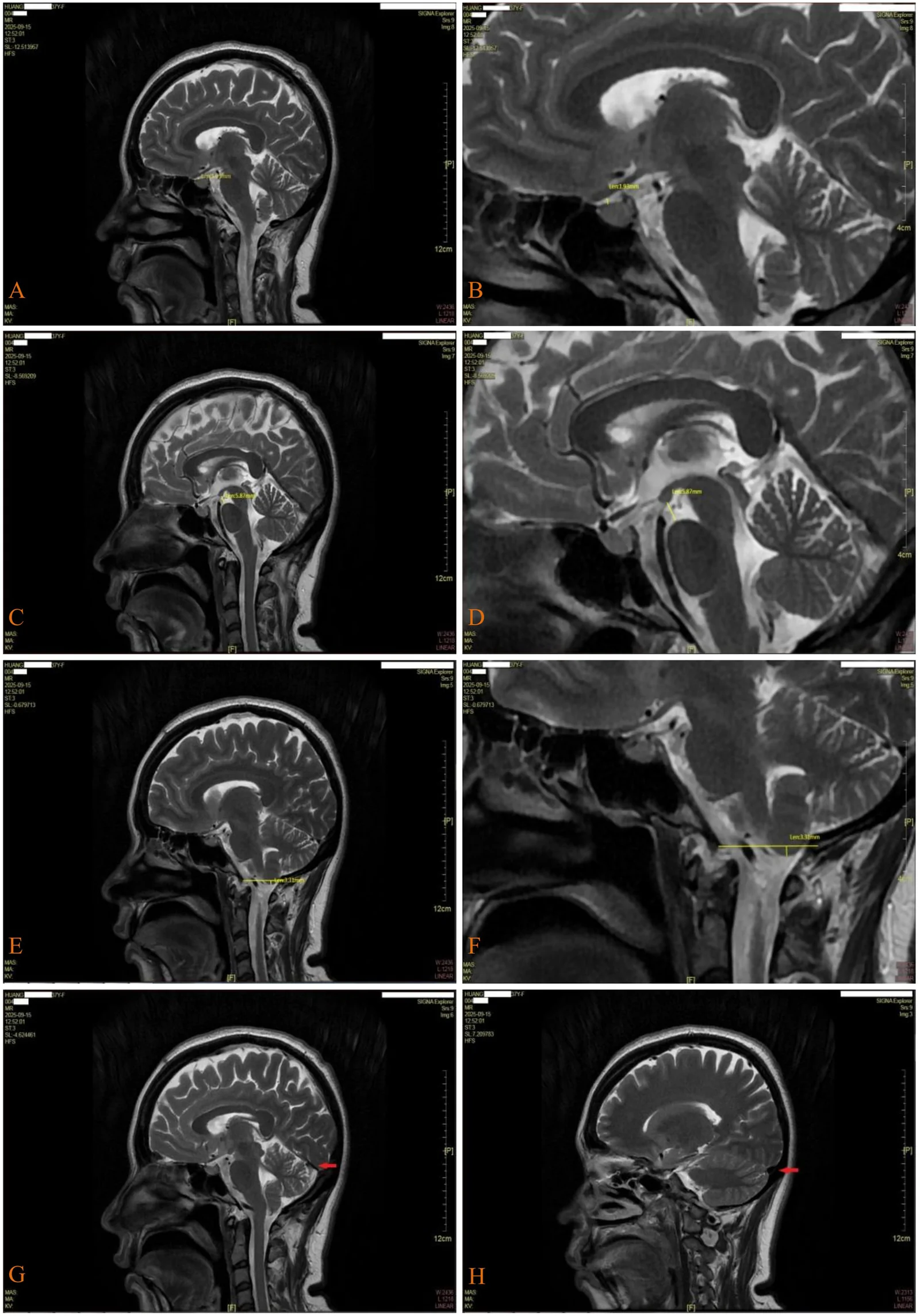
Initial cranial MRI showing imaging features consistent with intracranial hypotension. **(A)** Narrowing of the suprasellar cistern. **(B)** Magnified view of **(A)**. **(C)** Narrowing of the mamillopontine distance (approximately 5.8 mm; normal ≥6.5 mm). **(D)** Magnified view of **(C)**. **(E)** Inferior displacement of the cerebellar tonsils toward the foramen magnum. **(F)** Magnified view of **(E)**. **(G,H)** Mild dilatation of the venous sinuses with rounded morphology (red arrows). All image captures preserved original metadata including essential information and dates, while patient names and institutional details were redacted in compliance with privacy protection protocols.

**Table 2:** Comparison of cranial MRI measurements with normal values and follow-up values.

MRI parameter	Normal value	Initial MRI	Follow-up MRI (day 5)
Suprasellar cistern width (mm)	≥4	1.9	4.1
Mamillopontine distance (mm)	≥6.5	5.8	6.6
Cerebellar tonsillar position	At or above foramen magnum	Inferior displacement	Reduced inferior displacement
Venous sinus appearance	Normal caliber	Mild dilatation	Markedly improved

MRI, magnetic resonance imaging. Normal reference values are based on standard radiological criteria for intracranial hypotension.

Because of the high fever and leukocytosis, systemic infection could not be excluded, and EBP was deferred. Blood cultures were obtained on postpartum day 0 before empirical antibiotic therapy was initiated. The blood cultures subsequently showed no growth of bacteria, fungi, or anaerobes after 5 days; no other cultures were obtained, and no definite source of infection was identified. Despite initially normal CRP and procalcitonin, the high fever, leukocytosis, and a CRP rise to 40.1 mg/L at 48 h postpartum suggested a possible early or localized infection. After renewed informed consent, an initial non-blood-patch regimen was started: retained epidural catheter with continuous epidural normal saline at 10 mL/h; ceftriaxone 2 g every 12 h for a total of 5 days (fever resolved by 48 h postpartum, and antibiotics were continued to complete the 5-day course); diclofenac sodium suppository 50 mg rectally twice daily; strict bed rest; and intravenous fluid support. As the headache remained severe at 24 h postpartum, with a sitting NRS score of 10, intranasal dexmedetomidine was introduced at that time. A commercially available dexmedetomidine nasal spray [1 mL:500 μg; 25 μg per spray (15)] was used at a dose of 50 μg every 12 h, delivered as one spray into each nostril. Two doses were given in total, at 24 and 36 h postpartum. Blood pressure, heart rate, and oxygen saturation were monitored routinely throughout treatment. No hypotension, bradycardia, or excessive sedation was observed, and hemodynamic parameters remained stable (Table 1).

At 48 h postpartum, the patient had no headache in the supine position and tolerated gradual sitting at 45° and 90° for 5 min each without discomfort. The epidural catheter was removed. With complete headache resolution, intranasal dexmedetomidine was discontinued after only two doses (within 24 h). During the first postpartum day, the patient was unable to breastfeed because of severe headache and received formula feeding. After her headache improved on the second postpartum day, she resumed direct breastfeeding. On postpartum day 5, she had no significant discomfort during bed rest or ambulation, her neck was supple, and meningeal signs were negative. Follow-up cranial MRI showed improvement in the signs of intracranial hypotension, including widening of the suprasellar cistern and mamillopontine distance, reduced cerebellar tonsillar descent, and improvement of venous sinus dilatation (Figure 3; Table 2). She was discharged on postpartum day 6. No recurrence or related complications were reported at 1- and 3-month follow-up.

**Figure 3:**
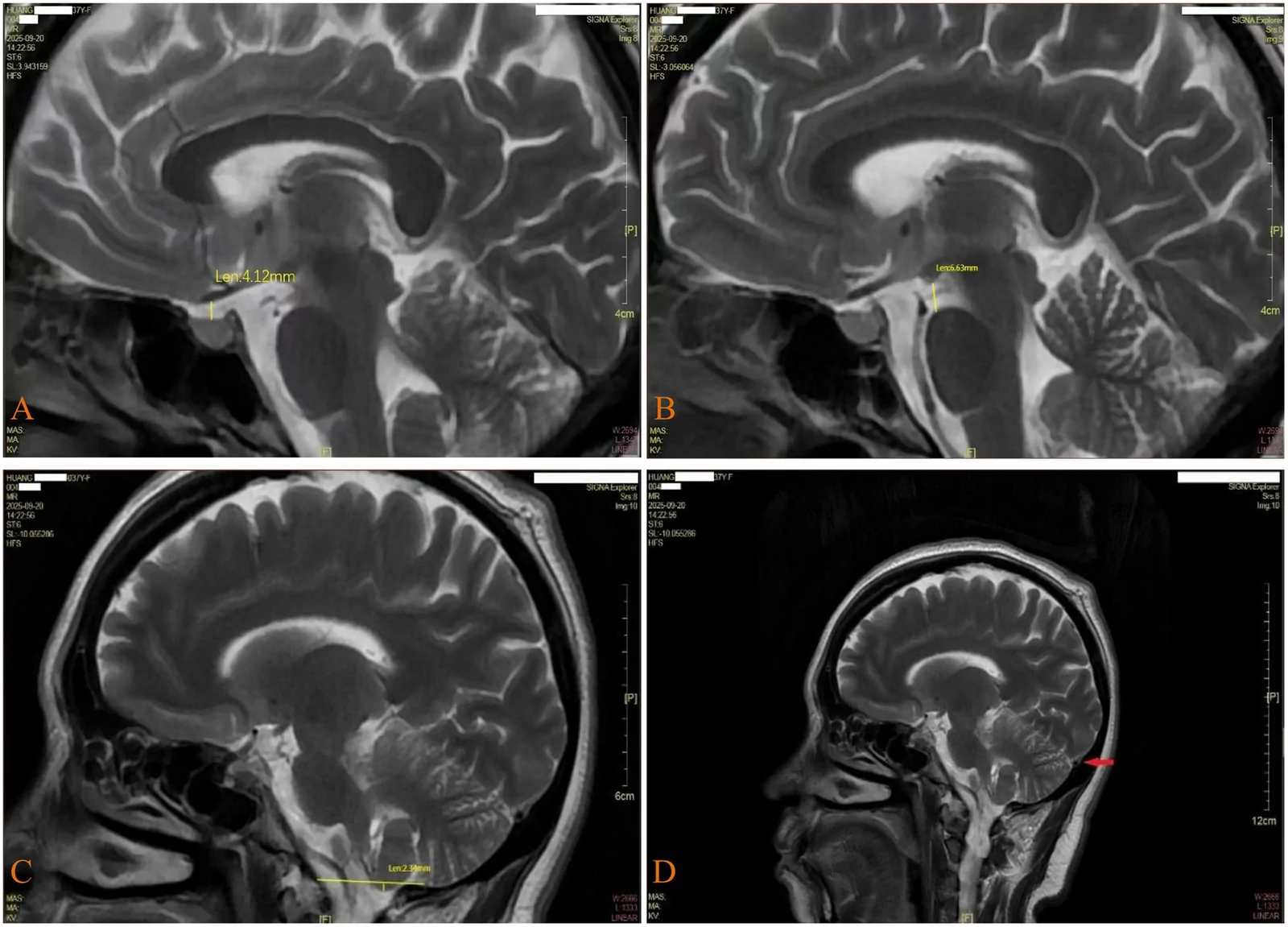
Follow-up cranial MRI on postpartum day 5 showing improvement in intracranial hypotension. **(A)** Widening of the suprasellar cistern (4.1 mm). **(B)** Widening of the mamillopontine distance (6.6 mm). **(C)** Upward displacement of the cerebellar tonsils. **(D)** Marked improvement of venous sinus dilatation (red arrow). All image captures preserved original metadata including essential information and dates, while patient names and institutional details were redacted in compliance with privacy protection protocols.

## Discussion

In patients with severe and persistent PDPH, clinicians should be alert to the possibility of intracranial hypotension. Most patients with PDPH improve gradually with conservative treatment; however, a small proportion may develop persistent CSF leakage, leading to severe intracranial hypotension and displacement of intracranial structures (16). In the present case, emergency cranial MRI demonstrated characteristic findings of intracranial hypotension: a narrowed suprasellar cistern (1.9 mm; normal ≥4 mm), a shortened mamillopontine distance (5.8 mm; normal ≥6.5 mm), inferior displacement of the cerebellar tonsils, and mild venous sinus dilatation (Table 2). Follow-up MRI on postpartum day 5 showed improvement in all these parameters, with the suprasellar cistern widening to 4.1 mm and the mamillopontine distance increasing to 6.6 mm, paralleling the patient's clinical recovery (Table 2). These objective imaging findings supported the timely diagnosis of intracranial hypotension syndrome and provided radiological evidence of treatment response (Figure 2). Such imaging documentation is relatively uncommon in PDPH case reports, many of which focus primarily on clinical symptoms rather than radiological confirmation of brain sagging or related structural changes.

For patients with severe PDPH in whom EBP is contraindicated or considered high risk, continuous epidural saline infusion through a catheter combined with intranasal dexmedetomidine may represent a feasible alternative strategy. The approach in this case is distinct from most previously reported non-EBP treatments, which have generally focused on single interventions (17). Katayama et al. and Liu et al. reported the use of continuous epidural saline infusion for PDPH (8, 18). However, epidural saline infusion alone may not provide rapid pain relief in all patients with severe PDPH, which is one reason EBP remains the preferred treatment in many cases (19). In the present case, although epidural saline infusion was initiated on the day of delivery, the patient had extremely poor sleep and persistent severe headache when sitting upright, with an NRS score of 10. Because EBP was considered high risk in the setting of fever and possible infection, a non-blood-patch adjunctive therapy was considered clinically important (20).

After the first dose of intranasal dexmedetomidine was administered at 24 h postpartum, the patient reported improved sleep and her headache decreased in a stepwise manner. The sitting NRS score decreased from 10 to 4 at 36 h postpartum and to 0 at 48 h postpartum. Dexmedetomidine may counteract compensatory intracranial vasodilation associated with PDPH through α2 receptor-mediated cerebral vasoconstriction. Its sedative and analgesic properties may also improve sleep quality and reduce psychological distress (14, 19). In a prospective randomized controlled trial, Mowafy et al. found that nebulized dexmedetomidine (1 μg/kg twice daily) significantly reduced visual analog scale scores in patients with PDPH, and transcranial Doppler showed a significant decrease in mean middle cerebral artery flow velocity (21). A recent systematic review and meta-analysis by Yang et al. further supported nebulized dexmedetomidine as a promising non-invasive treatment option for obstetric PDPH (22). PDPH is frequently self-limiting, and this patient received several treatments concurrently, including continuous epidural saline infusion, diclofenac, intravenous fluids, bed rest, and antibiotics. Accordingly, the observed clinical improvement cannot be assigned specifically to the saline–dexmedetomidine combination, and the independent contribution of dexmedetomidine remains uncertain (23). Nevertheless, headache severity declined progressively after intranasal dexmedetomidine was added, providing a rationale for further evaluation of this combined approach.

The intranasal route was selected because it is non-invasive, easy to administer, and does not require nebulization equipment (15). In this region, commercial intranasal dexmedetomidine formulations are available; reported bioavailability is approximately 59%, with generally favorable patient acceptability (15). Most published studies of dexmedetomidine for PDPH have used nebulized administration (13, 14, 24). Evidence from other clinical settings suggests that intranasal dexmedetomidine can provide a comparable sedative effect with good acceptance, and that nebulized administration is non-inferior to the intranasal route (25). Direct comparison of these delivery routes specifically in patients with PDPH is therefore warranted. In the present case, breastfeeding was resumed once the headache improved, which may have facilitated postpartum recovery and mother–infant bonding. Dodd et al. found very low concentrations of dexmedetomidine in breast milk after infusion (15–89 pg/mL), while Bethea et al. reported resumption of breastfeeding 60 min after anesthesia that included dexmedetomidine, without neonatal adverse events (26, 27). These observations are compatible with the absence of maternal or neonatal adverse effects in our patient, although they do not establish safety for all dosing regimens or routes of administration.

Compared with reports of nebulized dexmedetomidine alone, the combined strategy in this case appeared to be temporally associated with rapid headache resolution. Kavya et al. reported that after a single dose of nebulized dexmedetomidine, the median visual analog scale score at 24 h was 3 (interquartile range, 3–4), and the median score decreased to 0 at 48 h (24). In the present case, complete remission was observed 24 h after the first intranasal dexmedetomidine dose, or 48 h postpartum. Because this is an indirect comparison across different patients, routes, doses, and clinical contexts, it should be interpreted with caution. A potential explanation for the clinical response is that the two interventions act at different stages of the same pathophysiological process rather than through a simple additive effect. PDPH is primarily related to persistent CSF leakage, resulting in reduced intracranial pressure and compensatory cerebral vasodilation (23). Continuous epidural saline infusion may increase epidural pressure and promote closer apposition of the dura and arachnoid, thereby limiting CSF loss through the dural defect (10). Dexmedetomidine, a highly selective α2-adrenoceptor agonist, may influence a downstream component of this cascade by inducing α2-adrenoceptor-mediated cerebral vasoconstriction and thereby opposing compensatory vasodilation (22). Its sedative and analgesic actions may additionally improve sleep and lessen pain-related stress, factors that could further influence vascular responses (28). Because these treatments address distinct but related mechanisms—CSF leakage and secondary vasodilation—a complementary effect is biologically plausible. However, synergism cannot be inferred from a single case and should be tested in future studies using objective measures such as transcranial Doppler ultrasonography or serial MRI (21). Furthermore, previous randomized trials of dexmedetomidine for PDPH have not systematically evaluated sleep quality (13, 21, 24). In the present case, improved sleep after intranasal dexmedetomidine may have provided additional postpartum recovery benefits.

This case has several limitations. First, because of the urgent clinical circumstances, biochemical analysis of the fluid observed during puncture was not performed. Second, this is a single case report; therefore, causal inference is limited, and the efficacy and safety of the combined regimen require validation in larger prospective studies. Third, the patient's gestational diabetes mellitus and postpartum fever may have influenced the clinical course and treatment response. Fourth, most previous studies evaluated nebulized dexmedetomidine, whereas this case used intranasal dexmedetomidine; differences in bioavailability, onset time, and dose-response relationships require further investigation (29). Fifth, intranasal dexmedetomidine was administered for only 24 h, comprising two doses, because the patient's symptoms resolved rapidly. Thus, the optimal treatment duration cannot be determined from this case. In addition, validated instruments such as the Brief Pain Inventory, Headache Disability Index, and standardized sleep scales were not used. Although the patient's PDPH clearly interfered with breastfeeding and sleep during the early postpartum period, future studies should incorporate validated symptom, functional, and sleep assessments to characterize treatment response more comprehensively.

In conclusion, continuous epidural saline infusion combined with intranasal dexmedetomidine may be a feasible non-blood-patch option for selected patients with severe PDPH and intracranial hypotension when EBP is unsuitable or high risk. Because spontaneous improvement is common in PDPH and several interventions were administered simultaneously, this single case does not permit causal attribution of recovery to the combined regimen. Further prospective studies are required to clarify its efficacy, safety, and appropriate clinical use.

## Data Availability

The original contributions presented in the study are included in the article/Supplementary Material, further inquiries can be directed to the corresponding authors.
