# Supplementary material for: Continuous epidural saline infusion combined with intranasal dexmedetomidine for severe post-dural puncture headache with intracranial hypotension: a case report
Source: Front Med (Lausanne). 2026 Oct 26;13:1929098. doi: 10.3389/fmed.2026.1929098 (PMC13745558; doi:10.3389/fmed.2026.1929098)
Supplement: Supplementary file 1 [file Supplementaryfile1.pdf]

CARE Checklist of information to include when writing a case report

CARE case report guidelines

(cc) BY-NC-ND

Topic	Item	Checklist item description	Reported on Line
**Title**	1	The diagnosis or intervention of primary focus followed by the words "case report"	Title page
**Key Words**	2	2 to 5 key words that identify diagnoses or interventions in this case report, including "case report"	Abstract (Keywords)
**Abstract (no references)**	3a	Introduction: What is unique about this case and what does it add to the scientific literature?	Abstract (Backgroud)
	3b	Main symptoms and/or important clinical findings	Abstract (Case presentation)
	3c	The main diagnoses, therapeutic interventions, and outcomes	Abstract
	3d	Conclusion—What is the main "take-away" lesson(s) from this case?	Abstract
**Introduction**	4	One or two paragraphs summarizing why this case is unique (**may include references**)	Introduction
**Patient Information**	5a	De-identified patient specific information.	Case report
	5b	Primary concerns and symptoms of the patient.	Case report
	5c	Medical, family, and psycho-social history including relevant genetic information	Case report
	5d	Relevant past interventions with outcomes	Case report
**Clinical Findings**	6	Describe significant physical examination (PE) and important clinical findings.	Case report
**Timeline**	7	Historical and current information from this episode of care organized as a timeline	Figure 1; Table 1
**Diagnostic Assessment**	8a	Diagnostic testing (such as PE, laboratory testing, imaging, surveys).	Case report
	8b	Diagnostic challenges (such as access to testing, financial, or cultural)	Discussion
	8c	Diagnosis (including other diagnoses considered)	Case report
	8d	Prognosis (such as staging in oncology) where applicable	Not applicable
**Therapeutic Intervention**	9a	Types of therapeutic intervention (such as pharmacologic, surgical, preventive, self-care)	Case report
	9b	Administration of therapeutic intervention (such as dosage, strength, duration)	Case report
	9c	Changes in therapeutic intervention (with rationale)	Case report
**Follow-up and Outcomes**	10a	Clinician and patient-assessed outcomes (if available)	Case report
	10b	Important follow-up diagnostic and other test results	Case report
	10c	Intervention adherence and tolerability (How was this assessed?)	Discussion
	10d	Adverse and unanticipated events	Case report
**Discussion**	11a	A scientific discussion of the strengths AND limitations associated with this case report	Discussion
	11b	Discussion of the relevant medical literature **with references**.	Discussion
	11c	The scientific rationale for any conclusions (including assessment of possible causes)	Discussion
	11d	The primary "take-away" lessons of this case report (without references) in a one paragraph conclusion	Discussion
**Patient Perspective**	12	The patient should share their perspective in one to two paragraphs on the treatment(s) they received	Not applicable
**Informed Consent**	13	Did the patient give informed consent? Please provide if requested	Yes ☑ No ☐
